# Severe Pelvic Organ Prolapse Managed Without Surgery: Pessary Discontinued After Pelvic Floor Muscle Training With M‐Mode Ultrasound

**DOI:** 10.1002/iju5.70138

**Published:** 2026-01-07

**Authors:** Yukimasa Ide, Nobutaka Shimizu, Rio Ninomiya, Tomoko Ogawa, Tetsuya Fukumoto, Shinji Hyodo, Rie Yoshimura, Yoshitaka Kurano, Satoshi Fukata, Keiji Inoue

**Affiliations:** ^1^ Pelvic Floor Center, Kochi Medical School Kochi University Kochi Japan; ^2^ Department of Physical Therapy Yawatahama City General Hospital Ehime Japan; ^3^ Department of Occupational Therapy Yawatahama City General Hospital Ehime Japan; ^4^ Department of Urology Yawatahama City General Hospital Ehime Japan; ^5^ Department of Urology Graduate School of Medicine, Ehime University Ehime Japan; ^6^ Department of Obstetrics and Gynecology Yawatahama City General Hospital Ehime Japan; ^7^ Department of Urology Kochi Medical School, Kochi University Kochi Japan

**Keywords:** M‐mode ultrasonography, pelvic floor muscle training, pelvic organ prolapse, severe uterine prolapse, vaginal pessary

## Abstract

**Introduction:**

We report the case of a patient with severe uterine prolapse who underwent successful vaginal pessary removal after pelvic floor muscle training.

**Case Presentation:**

A 63‐year‐old woman presented with urinary dysfunction and residual urine. She was diagnosed with stage III pelvic organ prolapse by an obstetrician‐gynecologist, and a vaginal pessary was inserted. The patient underwent pelvic floor muscle training for 4 months while the vaginal pessary remained in situ. M‐mode ultrasonography revealed improved pelvic floor function, necessitating vaginal pessary removal. The patient's uterine prolapse improved to pelvic organ prolapse‐quantification stage II without recurrence of pelvic organ prolapse, urinary dysfunction, or residual urine after 2 years.

**Conclusions:**

In patients with severe uterine prolapse who use a vaginal pessary, appropriate pelvic floor muscle training guided by a physical therapist may eliminate the need for continued pessary use.

## Keynote Message

Pelvic floor muscle training (PFMT) guided by a physical therapist using M‐mode ultrasonography enhances pelvic floor muscle function, reduces the duration of bladder base elevation (BBE), and increases BBE speed in patients with severe uterine prolapse during vaginal pessary insertion. For patients seeking pessary removal and preferring nonsurgical intervention, continued appropriate PFMT may facilitate ring pessary removal.

**Abbreviations**
BBE: bladder base elevation
MRI: magnetic resonance imaging
PFM: pelvic floor muscles
PFMT: pelvic floor muscle training
POP: pelvic organ prolapse
POP‐Q: pelvic organ prolapse‐quantification
PT: physical therapist
UI: urinary incontinence

## 1 Introduction

Pelvic organ prolapse (POP) affects approximately 40% of women. POP prevalence may increase with an aging population [1]. The most commonly reported risk factors for POP are aging, childbirth, and obesity [2]. Treatment options include pelvic floor muscle training (PFMT), surgery, and vaginal pessaries [1]. PFMT is the first‐line treatment for mild POP (pelvic organ prolapse‐quantification [POP‐Q] stage I/II) [2, 3]. However, it is less frequently implemented in severe cases (POP‐Q stages III/IV) [4]. Reports of successful pessary removal using PFMT remain limited. M‐mode ultrasonography measures bladder base elevation (BBE) during PFMT. It provides reproducible indices such as BBE time and speed [5, 6]. The M‐mode assessment is a key feature of the proposed approach.

We present a case in which a pessary was successfully removed from a patient with severe POP under PFMT guidance.

## 2 Case Presentation

**FIGURE 1:**
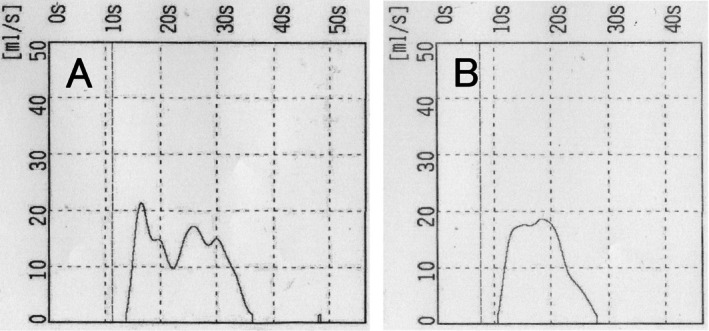
Uroflowmetry before (pessary in situ) and after ring pessary removal. Pre‐insertion measurements were not obtained. (A) Vaginal pessary insertion (urine volume: 288.4 mL; maximum urinary flow rate: 21.4 mL/s, average urinary flow rate: 12.5 mL/s, and residual urine: 0 mL). (B) After vaginal pessary removal (urine volume: 217.3 mL, maximum urinary flow rate: 18.7 mL/s, average urinary flow rate: 12.6 mL/s, and residual urine: 0 mL).

A 63‐year‐old woman (height: 151 cm; weight: 57 kg; body mass index: 25 kg/m^2^) presented with dysuria 18 months before PFMT initiation. She was diagnosed with POP‐Q stage III uterine prolapse by a gynecologist, and a vaginal pessary was inserted. The gynecologist documented only the POP‐Q stage (stage III at baseline, improved to stage II); however, detailed POP‐Q point measurements (Aa, Ba, and C) were not recorded. Although the vaginal pessary improved urinary dysfunction (Figure 1A), the patient requested its removal. PFMT was initiated with a physical therapist (PT) while the pessary remained in place. Pelvic floor function was evaluated using M‐mode ultrasonography and cine MRI.

Transabdominal ultrasonography was used to visualize the bladder in the mid‐sagittal plane during urine storage. The time and speed of BBE during PFMT were measured using M‐mode ultrasonography (the average of three measurements was used) [5]. BBE time was defined as the time from the onset of bladder base elevation caused by pelvic floor muscle contraction to the maximal elevation on M‐mode ultrasonography. BBE speed (mm/s) was calculated by dividing bladder base elevation distance (mm) measured on the same M‐mode image by BBE time (s) (Aplio 300 system, Canon Medical Systems Corporation). A 3.5‐MHz convex‐type probe was used with the patient in the supine position, with the hip and knee joints flexed slightly.

**FIGURE 2:**
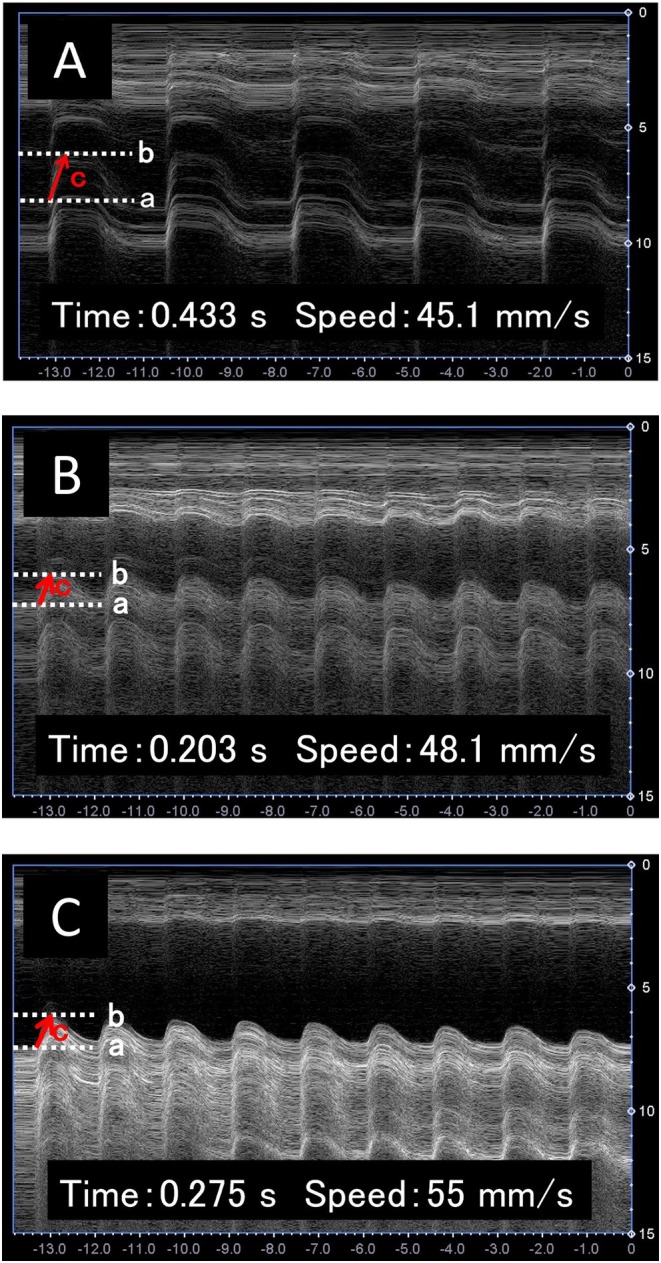
Alterations in bladder‐base elevation waveform are observed through M‐mode ultrasonography during pelvic floor muscle training (PFMT). a: Baseline of the bladder base; b: Maximum elevation line; c: Displacement used for quantitative measurements. (A) During PT intervention (bladder base elevation time, 0.433 s; bladder base elevation speed: 45.1 mm/s). (B). After four months of continued PFMT (bladder base elevation time, 0.203 s; bladder base elevation speed: 48.1 mm/s). (C) After vaginal pessary removal (bladder base elevation time, 0.275 s; bladder base elevation speed, 55 mm/s).

Reference values (BBE time: 0.186 s; BBE speed: 26.5 mm/s) were derived from five healthy women in their 20s for comparative analysis. Upon initiating PFMT, the BBE time and speed were recorded as 0.433 s and 45.1 mm/s, respectively (Figure 2A), indicating a slower BBE time and prolonged PFM contraction compared to the healthy female cohort.

PFMT included coordinated training of the trunk, pelvic floor, and hip joints.

The PFMT program included nine supervised 60‐min sessions and a structured home regimen of more than 100 daily PFM contractions (average, 300), with full adherence for 4 months.

**FIGURE 3:**
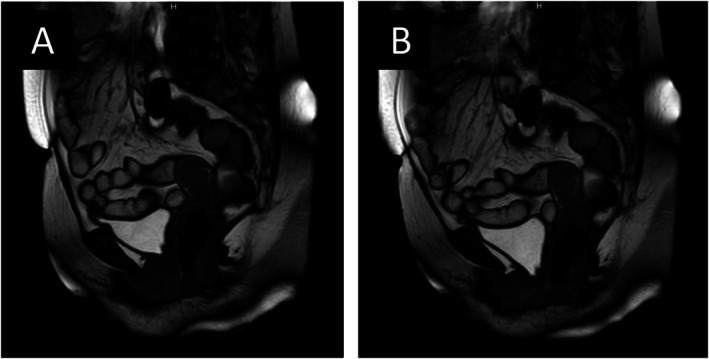
Cine magnetic resonance imaging (MRI) images depict the position of pelvic organs after pessary removal. No pre‐insertion MRI data were available. (A) Cine MRI at rest. (B) Cine MRI with maximum abdominal pressure.

Initially, PFM contraction was suboptimal in sitting and standing positions. Thus, PFMT was performed in the supine position for the first month, with sitting and standing introduced after 2 months. All self‐training activities were documented in the PFMT log sheet. No specific lifestyle modification program (other than PFMT) for weight reduction or intra‐abdominal pressure management was provided. After 4 months, the BBE time decreased significantly from 0.433 to 0.203 s, and the BBE speed increased slightly from 45.1 to 48.1 mm/s (Figure 2B), suggesting improved PFM function and prompting pessary removal. After pessary removal, the BBE time and speed were 0.275 s and 55 mm/s, respectively (Figure 2C). This reflected a slight increase in BBE time compared with the pre‐removal values, but an improvement from POP‐Q stage III to II (Figure 3). Cine MRI was performed 4 months after PFMT and pessary removal. Relative to the pubococcygeal line, pre‐removal (pessary in situ), the bladder neck was +6 mm at rest and −16 mm on straining; post‐removal, it was +4 mm at rest and −17 mm on straining, indicating only marginal additional descent. No urinary dysfunction (Figure 1B) or residual urine loss occurred; the urinary symptoms were not exacerbated. The patient maintained stable POP and urinary symptoms for 2 years without a pessary, with no deterioration observed on gynecological examination, and cine MRI around the time of pessary removal showed no marked worsening of pelvic organ descent.

## 3 Discussion

In patients with POP, PFMT performed while sitting or standing can induce compensatory movements because the pelvic organs are insufficiently supported. Therefore, PFMT was initially performed in the supine position to reduce the pelvic floor load and ensure proper contraction learning. The pessary also provides temporary support to facilitate effective PFMT. This combined approach may be appropriate for patients with severe POP who prefer conservative management and supervised training.

In POP‐Q stage II vaginal prolapse, combining a pessary with PFMT may show greater therapeutic benefits than PFMT alone [7].

Continued PFMT, while the vaginal pessary was in place, reduced BBE time from 0.433 to 0.203 s in this patient with severe POP. Previously, we documented that PFMT decreased BBE time and improved urinary incontinence (UI) in patients with UI following radical prostatectomy [5] and identified a correlation between BBE time and UI volume [8].

The reduction in BBE duration from 0.433 to 0.203 s was accompanied by POP‐Q stage improvement, suggesting a potential correlation between BBE duration and POP amelioration. The BBE time (onset‐to‐peak in M‐mode) likely shortens with faster neuromuscular recruitment and improved coordination of the pelvic floor and synergistic trunk musculature, indicating greater contractile efficiency. Tonic activation and endurance of slow‐twitch fibers, which stabilize the pelvic floor and help maintain an elevated posture, may reduce the required excursion and further reduce time‐to‐peak. A shorter BBE time may enable more timely elevation/support during intra‐abdominal pressure surges, contributing to functional improvements in prolapse management. However, BBE velocity exhibited only a marginal increase. This is possibly because the pelvic floor became less lax (owing to the transition from POP‐Q stage III to II) and was elevated by PFMT, thereby reducing BBE length.

The continuation of PFMT is likely facilitated by structured patient education and a self‐monitoring log. Recent studies have demonstrated that strategies such as goal setting, follow‐up, and exercise diaries can enhance the adherence to and long‐term efficacy of PFMT [9, 10].

This case report had some limitations. First, it describes a single case, which restricts its generalizability. Second, the reproducibility of M‐mode ultrasonography for pelvic floor assessment remains unclear. Our reference values were established based on healthy women in their 20s rather than on those of age‐matched controls, underscoring the need for age‐stratified norms. Additional studies with larger sample sizes are required to validate our findings. No recurrence occurred over 2 years with continued voluntary PFMT, even after pessary removal.

## 4 Conclusion

Structured, sustained, and supervised PFMT may prevent the recurrence of severe POP, enable sustained functional recovery, and complete cessation of pessary use, even in patients with severe uterine prolapse requiring a vaginal pessary.

## Ethics Statement

The study protocol was approved by the Institutional Ethics Committee and conformed to the provisions of the Declaration of Helsinki (Yawatahama City General Hospital Committee, Approval No. 20230502–002).

## Consent

Written informed consent was obtained from the patient.

## Conflicts of Interest

The authors declare no conflicts of interest.

## Data Availability

The data that support the findings of this study are available from the corresponding author upon reasonable request.
